# Primary headache drug treatment in emergency departments in Australia and New Zealand

**DOI:** 10.5694/mja2.51670

**Published:** 2022-08-02

**Authors:** Kevin Chu, Anne‐Maree Kelly, Frances Kinnear, Gerben Keijzers, Sinan Kamona

**Affiliations:** ^1^ The University of Queensland Brisbane QLD; ^2^ Royal Brisbane and Women's Hospital Brisbane QLD; ^3^ Joseph Epstein Centre for Emergency Medicine Research, Western Health Melbourne VIC; ^4^ Gold Coast University Hospital Gold Coast QLD; ^5^ Auckland City Hospital Grafton New Zealand

**Keywords:** Headache, Migraine, Prescribing, Guidelines as topic, Emergency treatment

Treating acute headache begins at home and continues, if necessary, in the emergency department (ED). Drug treatment is heterogenous,^1^ and understanding the determinants of prescribing could help appropriate medication choices. We therefore assessed the predictors of prescribing in Australian and New Zealand EDs for people with primary headache.

We undertook a secondary analysis of Australian and New Zealand data from the multinational HEAD study (Supporting Information).^2^ The major outcome was drug administration to people presenting to EDs with primary headache. We assessed the use of paracetamol, non‐steroidal anti‐inflammatory drugs (NSAIDs), opioids, anti‐dopaminergic agents (prochlorperazine, metoclopramide, droperidol), ondansetron, chlorpromazine (intravenous infusion), and triptans. We included headache characteristics and pre‐hospital medications as fixed effect predictors in a multilevel binary logistic regression model; hospital group was included as a random effect to account for clustering of patients within groups. The analysis was conducted in Stata 16.1. The Melbourne Health Human Research Ethics Committee approved the HEAD study (HREC/43148/MH‐2018), which was prospectively registered with the Australian New Zealand Clinical Trials Registry (ACTRN12619000094178).

A total of 1521 people with primary headache attended the 44 participating EDs (median age, 41 years [interquartile range, 29–55 years]; 1102 women [72%]; 382 transported by ambulance [25%]) (Supporting Information, table 1). Prior to arrival, 600 had self‐medicated with non‐opioid analgesics (39%) and 177 with opioids (codeine, oxycodone, tramadol) (12%). Ambulance staff had provided non‐opioid analgesics to 94 of 382 patients (25%), opioids to 96 (25%; most frequently fentanyl [60, 16%] or morphine [31, 8%]), and anti‐emetics to 139 (36%) (Supporting Information, table 2).

In the EDs, non‐opioid analgesics were administered to 1036 people (68%), opioids to 495 (33%; most frequently oxycodone [224, 15%] or codeine [214, 14%]), anti‐dopaminergic agents to 478 (31%), ondansetron to 321 (21%), chlorpromazine (infused) to 281 (18%), and triptans to 46 patients (3%) (Supporting Information, table 3). Eight treatments accounted for 51% of the 88 drug combinations used in the ED (no drug, 17%; paracetamol alone, 8%; paracetamol/NSAID, 6%; paracetamol/NSAID/anti‐dopaminergic, 5%; paracetamol/NSAID/opioid, paracetamol/opioid, or opioid only, each 4%; paracetamol/anti‐dopaminergic, 3%).

Opioids were more frequently provided in the ED to people with moderate or severe rather than mild headache, those with prolonged headache (duration longer than three days), and people who used an opioid before arriving at the ED (Box). Other drugs were used in combination with an opioid in 464 of 495 cases (94%), but not a guideline‐recommended anti‐migraine medication (anti‐dopaminergic, chlorpromazine, triptan) in 240 of 495 cases (48%). Ondansetron, without proven anti‐migraine effects, was given more frequently than an anti‐dopaminergic to relieve nausea or vomiting (Box).

Box 1 **Drugs given to people presenting with primary headache to 44 emergency departments in Australia and New Zealand: multilevel binary logistic regression analysis (odds ratios with 95% confidence intervals)**

**Table:** 

Fixed effect predictors	Paracetamol	NSAIDs	Opioids	Anti‐dopaminergics*	Chlorpromazine^†^	Ondansetron	Triptans
Age, per 10 years	0.98 (0.92–1.04)	**0.79** **(0.74–0.85)**	1.00 (0.94–1.08)	0.88 (0.82–0.95)	0.94 (0.85–1.04)	0.90 (0.82–0.98)	0.93 (0.76–1.14)
Sex (men)	0.88 (0.69–1.12)	1.05 (0.82–1.35)	1.09 (0.84–1.41)	**0.72** **(0.54–96)**	0.78 (0.55–1.13)	0.84 (0.60–1.16)	1.01 (0.49–2.06)
History of migraine	0.96 (0.76–1.22)	**1.44** **(1.14–1.83)**	1.05 (0.81–1.34)	**1.90** **(1.46–2.47)**	**2.80** **(2.04–3.85)**	**1.48** **(1.11–1.98)**	**2.33** **(1.21–4.46)**
Headache severity, (*v* mild)							
Moderate	**1.95 (1.34–2.82)**	**2.32** **(1.55–3.48)**	**2.09** **(1.31–3.33)**	1.34 (0.84–2.16)	**3.59** **(1.73–7.44)**	1.28 (0.71–2.32)	1.57 (0.44–5.53)
Severe	**2.20** **(1.51–3.22)**	**2.62** **(1.74–3.95)**	**3.13** **(1.96–4.98)**	**2.02** **(1.26–3.24)**	**4.30** **(2.07–8.95)**	**2.76** **(1.55–4.91)**	1.85 (0.53–6.48)
Unknown	1.29 (0.86–1.95)	**1.73** **(1.10–2.71)**	1.66 (1.00–2.77)	**1.73** **(1.02–2.93)**	2.30 (1.04–5.05)	1.58 (0.84–2.97)	0.89 (0.21–3.89)
Headache duration (*v* < 1 day)							
1–3 days	1.29 (0.97–1.72)	**1.44** **(1.08–1.92)**	1.05 (0.78–1.43)	0.88 (0.64–1.23)	0.89 (0.59–1.35)	1.07 (0.76–1.51)	1.05 (0.48–2.30)
> 3 days	1.12 (0.87–1.43)	0.98 (0.76–1.26)	**1.37** **(1.05–1.78)**	1.16 (0.87–1.55)	**1.72** **(1.20–2.45)**	**0.65** **(0.46–0.91)**	1.01 (0.49–2.07)
Unknown	0.70 (0.29–1.66)	0.64 (0.26–1.60)	0.72 (0.26–2.03)	0.64 (0.22–1.87)	1.43 (0.45–4.57)	0.51 (0.14–1.90)	1.43 (0.17–11.7)
Nausea/vomiting	**1.32** **(1.05–1.67)**	1.22 (0.97–1.54)	1.28 (1.00–1.63)	**2.15** **(1.66–2.79)**	**2.02** **(1.45–2.81)**	**4.11** **(2.99–5.64)**	1.22 (0.63–2.37)
Pre‐hospital drugs taken							
Paracetamol	**0.55** **(0.43–0.69)**	1.0 (0.79–1.26)	1.20 (0.93–1.53)	0.99 (0.76–1.30)	1.01 (0.73–1.41)	0.99 (0.74–1.34)	0.69 (0.35–1.37)
Non‐steroidal anti‐inflammatory drug	0.89 (0.67–1.18)	0.80 (0.59–1.07)	1.06 (0.78–1.43)	1.03 (0.75–1.43)	1.32 (0.90–1.94)	0.97 (0.68–1.40)	1.52 (0.73–3.16)
Opioid	0.95 (0.70–1.28)	1.06 (0.79–1.44)	**1.46** **(1.08–1.98)**	1.32 (0.95–1.84)	**1.78** **(1.22–2.59)**	1.02 (0.72–1.46)	1.29 (0.61–2.72)
Anti‐emetic	0.71 (0.50–1.0)	0.56 (0.39–0.81)	0.82 (0.57–1.17)	0.98 (0.67–1.43)	0.91 (0.59–1.41)	0.72 (0.48–1.08)	0.95 (0.38–2.35)
Triptan	1.20 (0.74–1.96)	0.94 (0.58–1.51)	0.76 (0.45–1.27)	0.98 (0.59–1.64)	1.74 (0.99–3.07)	0.88 (0.51–1.54)	1.16 (0.41–3.27)

* Prochlorperazine, metoclopramide, droperidol.

† Unlike other anti‐dopaminergic agents, chlorpromazine is not typically used as an anti‐emetic, but given as an intravenous infusion for treating intractable migraine.^3^ Bold: confidence interval does not include 1.0.

Chlorpromazine was more frequently provided in the ED to people with severe headache than other drugs (Box); it was administered as a second drug rather than as the initial drug in 160 of 281 cases (57%; Supporting Information, table 3), as part of a stepped care approach. Stratified care, in which initial treatment is based on headache severity and disability, may provide greater headache relief,^4^ but this has not been confirmed in ED care.

Triptans were given infrequently in the ED, usually to people with a history of migraine (Box), possibly because triptans are regarded as effective only when taken early in headache onset. Subcutaneous sumatriptan, however, is recommended by Australian guidelines for intractable migraine (longer than 72 hours)^3^ and by the American Headache Society for eligible migraineurs seeking help in the ED.^5^

Our findings suggest that evidence‐base recommendations are not always followed in the ED, but do not provide information about the most effective treatment options. ED practice changes to better reflect guideline recommendations could include using parenteral prochlorperazine or metoclopramide rather than ondansetron in people with primary headache pain and vomiting,^5^ improving hospital opioid stewardship to avoid overuse of opioids,^6^, ^7^ providing subcutaneous triptans to eligible migraineurs,^3^, ^5^ and considering subcutaneous triptans or intravenous chlorpromazine as initial treatment options for moderate to severe migraine in a stratified care approach.^4^

## Open access

Open access publishing facilitated by The University of Queensland, as part of the Wiley – The University of Queensland agreement via the Council of Australian University Librarians.

## Competing interests

No relevant disclosures.

## Supporting information


Appendix S1.
