# Supplementary material for: Primary headache drug treatment in emergency departments in Australia and New Zealand
Source: Med J Aust. 2022 Aug 2;217(7):366–7. doi: 10.5694/mja2.51670 (PMC9804343; doi:10.5694/mja2.51670)
Supplement: Supplementary file 1 — Appendix S1. [file MJA2-217-366-s001.pdf]

The Medical Journal of Australia

Supporting Information

Supplementary methods and results

This appendix was part of the submitted manuscript and has been peer reviewed.
It is posted as supplied by the authors.

Appendix to: Chu K, Kelly AM, Kinnear F, et al. Primary headache drug treatment in emergency departments in Australia and New Zealand. *Med J Aust* 2022; doi: 10.5694/mja2.51670.

The HEAD study

The HEAD study was a prospective observational study of emergency department (ED) headache presentations conducted over one month, mostly March, in ten countries during 2019.[1,2] The study we report in this article was a secondary analysis of the Australian and New Zealand data from the HEAD study. The analysis included 44 EDs: 13 in Victoria, nine in New Zealand, eight in New South Wales, five in Queensland, four in South Australia, three in Western Australia, and two in the Northern Territory. EDs were selected from our pre-existing research network, mostly public hospitals across metropolitan and regional centres. Consecutive patients aged 18 years or more with non-traumatic headache as the primary presenting complaint were included. Inter-hospital transfers and re-presentations were excluded. Patients were included in this study if they had a final ED diagnosis of migraine, tension-type, cluster, musculoskeletal, or primary headache not otherwise specified.

1. Kelly AM, Kuan WS, Chu KH, et al. Epidemiology, investigation, management, and outcome of headache in emergency departments (HEAD study): a multinational observational study. *Headache* 2021; 61: 1539-1552.
2. Kelly AM, Chu K. Protocol, head study, headache in Emergency Departments 2018. https://www.anzctr.org.au/Steps11and12/376695-(Uploaded-10-01-2019-12-48-42)-Study-related%20document.pdf (viewed June 2022).

HEAD Study Steering Group

Anne Maree Kelly; Kevin Chu; Win Sen Kuan; Gerben Keijzers; Frances Kinnear; Mehmet Karamercan; Sharon Klim; Tissa Wijeratne; Sinan Kamona; ColinGraham; Richard Body; Tom Roberts; Daniel Horner; Said Laribi

HEAD study group

Catherine Lunter (Coffs Harbour Hospital, New South Wales), Rochelle Facer (Concord Repatriation Hospital, New South Wales), David Thomson (Port Macquarie Base and Kempsey District Hospitals, New South Wales), Robert Day (Royal North Shore Hospital, New South Wales), Greg McDonald (Sydney Adventist Hospital, New South Wales), Sarah Jones (Tamworth Regional Hospital, New South Wales), Julian Cochrane (Orange Base Hospital, New South Wales), Stephen Gourley (Alice Springs Hospital, Northern Territory), Mark Ross and Vinay Gangathimmaiah (Royal Darwin Hospital, Northern Territory), Kim Hansen (St Andrew's War Memorial Hospital, Queensland), Frances Kinnear (The Prince Charles Hospital, Queensland), Gerben Keijzers (Gold Coast University Hospital, Queensland), Kevin Chu and Nathan Brown (Royal Brisbane and Women's Hospital, Queensland), Paul Bowe (Robina Hospital, Queensland), Raymund de la Cruz (Lyell McEwin and Modbury Hospitals, South Australia), Daniel Haustead (The Queen Elizabeth and Royal Adelaide Hospitals, South Australia), Jean Moller (University Hospital Geelong, Victoria), Katie Walker (Cabrini Malvern, Victoria), Richard Smith (Bendigo Health, Victoria), Ron Sultana (Epworth Healthcare, Victoria), John Pasco (Werribee Mercy Hospital, Victoria), Neil Goldie and Andis Graudins (Monash Health, Victoria), Rosamond Dwyer (Peninsula Health, Victoria), George Plunkett (Melbourne Health, Victoria), Anne-Maree Kelly (Western Health, Victoria), Hugh Mitenko (WA Country Health Service, Western Australia), Michael Lovegrove (Joondalup Health Campus, Western Australia), Ben Smedley (Rockingham General Hospital, Western Australia), Colin A Graham and Ling Yan Leung (Prince of Wales Hospital, Hong Kong SAR), Win Sen Kuan and Ying Wei Yau (National University Hospital, Singapore), Wei Ming Ng (Ng Teng Fong General Hospital, Singapore), Ranjeev Kumar (Khoo Teck

Puat Hospital, Singapore), Dennis Wen Jie Chia (Sengkang General Hospital, Singapore), Said Laribi (CHU Tours, Tours, France), Mounir Hilal and Rarthtana Mil (CH Vendôme, France), Audrey Gerineau (CHR Orléans, France), Matthew Reed (Emergency Medicine Research Group Edinburgh [EMERGE], Royal Infirmary of Edinburgh, United Kingdom), Daniel Horner (Salford Royal NHS Foundation Trust, Salford, United Kingdom), Edward Carlton and Tom Roberts (North Bristol NHS Trust, United Kingdom), Girish Boggaram and Jayne Foot (Musgrove Park Hospital, Taunton, United Kingdom), Andy Appleboam, Rachel Goss and Hamza Malik (Royal Devon and Exeter NHS Foundation, United Kingdom), Richard Body (Manchester Royal Infirmary, Manchester, United Kingdom), John-Paul Williamson (Royal Oldham Hospital, Oldham, United Kingdom), Adela Golea and Sonia Luka (University County Hospital Cluj-Napoca, Romania), Huseyin Avni Demir (University of Health Sciences Mehmet Akif Inan Training and Research Hospital, Department of Emergency Medicine, Şanlıurfa Turkey), Şafak Öner Gülpinar (Tokat Erbaa Government Hospital, Tokat, Turkey), Lale Tolu (Bursa Çekirge Government Hospital of Emergency Service, Bursa, Turkey), Muhammet Hacimustafaoğlu (Hakkari Yuksekova Government Hospital, Hakkari, Turkey), Mehmet Karamercan (Gazi University Faculty of Medicine Department of Emergency Medicine, Ankara, Turkey), Elif Çelikel (Numune Research and Training Hospital, Department of Emergency Medicine, Ankara, Turkey), Çilem Çaltili (Unıversıty of Health Sciences Bağcılar Training and Research Hospital, Department of Emergency Medicine, Istanbul, Turkey), Selahattin Gürü (Yıldırım Beyazıt University Faculty of Medicine Department of Emergency Medicine, Ankara, Turkey), Gülşah Yavuz (Antalya Ataturk Government Hospital of Medicine, Department of Emergency Medicine, Antalya, Turkey), Franck Verschuren (Institute of Experimental and Clinical Research, Emergency Department, Saint-Luc University Hospital, Brussels, Belgium), Christopher Ramos (Emergency Department, Saint-Luc University Hospital, Brussels, Belgium), Paule Denoel and Nicolas Wilmet (Saint Michel, Clinique de l'Europe, Etterbeek, Brussels), Michael Vandoorslaert and Alessandro Manara (Saint Elisabeth, Clinique de l'Europe, Uccle, Brussels), Adeline Higuet (CHR Hal, Belgium), Amichai Sheffy (Tel-Aviv Sourasky Medical Center, Israel), Sinan Kamona and Peter Jones (University of Auckland, School of Medicine, Auckland, New Zealand), Mai Nguyen (Wellington Hospital, Wellington, New Zealand), Anne Clarke (Hutt Valley Hospital, Lower Hutt, New Zealand), Sierra Beck (Dunedin Hospital, Dunedin, New Zealand), Andrew Munro (Nelson Hospital, Nelson, New Zealand), Kim Yates (North Shore and Waitakere Hospitals, Waitematā District Health Board, New Zealand), James Weaver (Christchurch Hospital, Christchurch, New Zealand), Deborah Moore and Stuart Innes (Tauranga Hospital, Tauranga, New Zealand), Karina Walters (Taranaki District Health Board, New Zealand). Co-ordinating Centre, Western Health, Victoria: Anne-Maree Kelly, Sharon Klim and Kerrie Russell.

Table 1. Characteristics of 1521 emergency department patients with primary headache

Characteristic	All patients	Non-ambulance patients	Ambulance patients
Patients	1521	1139 [75%]	382 [25%]
Age (years)			
Median (interquartile range)	41 (29–55)	39 (28–53)	46 (35–62)
> 50 years	481 (32%)	325 (29%)	156 (41%)
Sex (women)	1102 (72%)	821 (72%)	281 (74%)
Referred by			
Self	1272 (84%)	943 (83%)	329 (86%)
General practitioner	249 (16%)	196 (17%)	53 (14%)
Australasian Triage Scale category			
1	2 (0.1%)	1 (0.1%)	1 (0.3%)
2-3	942 (62%)	692 (61%)	250 (65%)
4-5	577 (38%)	446 (39%)	131 (34%)
Headache severity			
Mild	163 (11%)	125 (11%)	38 (10%)
Moderate	539 (35%)	424 (37%)	115 (30%)
Severe	546 (36%)	388 (34%)	158 (41%)
Unknown	273 (18%)	202 (18%)	71 (19%)
Headache duration			
< 1 day	703 (46%)	437 (38%)	266 (70%)
1-3 days	308 (20%)	251 (22%)	57 (15%)
> 3 days	486 (32%)	430 (38%)	56 (15%)
Unknown	24 (2%)	21 (2%)	3 (0.8%)
Nausea or vomiting	774 (51%)	573 (50%)	201 (53%)
Headache diagnosis			
Primary not further specified	703 (46%)	520 (46%)	183 (48%)
Migraine	684 (45%)	516 (45%)	168 (44%)
Tension-type	93 (6%)	74 (6%)	19 (5%)
Musculoskeletal	21 (1%)	15 (1%)	6 (2%)
Cluster	20 (1%)	14 (1%)	6 (2%)
Disposition			
Discharged from short-stay unit	874 (57%)	674 (59%)	200 (52%)
Discharged from emergency department	534 (35%)	385 (34%)	149 (39%)
Admitted to hospital inpatient unit	108 (7%)	76 (7%)	32 (8%)
Unknown	5 (0.3%)	4 (0.4%)	1 (0.3%)

Table 2. Pre-hospital drug treatment of primary headache

**Drug**	**All patients**	**Non-ambulance patients**	**Ambulance patients**
Patients	1521	1139	382
Self-medicated by patient			
Non-opioid analgesics	600 (39%)	465 (41%)	135 (35%)
Paracetamol	507 (33%)	385 (34%)	122 (32%)
NSAIDs	284 (19%)	239 (21%)	45 (12%)
Opioid analgesics, any	177 (12%)	140 (12%)	37 (10%)
Codeine	109 (7%)	87 (8%)	22 (6%)
Oxycodone	41 (3%)	31 (3%)	10 (3%)
Tramadol	32 (2%)	26 (2%)	6 (2%)
Other opioids	15 (1%)	10 (0.9%)	5 (1%)
Anti-emetics	68 (4%)	48 (4%)	20 (5%)
Triptans	86 (6%)	68 (6%)	18 (5%)
Any of the above self-medicated drugs	721 (47%)	555 (49%)	166 (43%)
Given by ambulance service			
Non-opioid analgesics	94 (6%)	N/A	94 (25%)
Paracetamol	89 (6%)	N/A	89 (23%)
NSAIDs	11 (0.7%)	N/A	11 (3%)
Opioid analgesics, any	96 (6%)	N/A	96 (25%)
Fentanyl	60 (4%)	N/A	60 (16%)
Morphine	31 (2%)	N/A	31 (8%)
Other opioids	7 (0.5%)	N/A	7 (2%)
Anti-emetics	139 (9%)	N/A	139 (36%)
Triptans	2 (0.1%)	N/A	2 (0.5%)
Methoxyflurane	25 (2%)	N/A	25 (7%)
Any of the above ambulance drugs other than methoxyflurane	217 (14%)		217 (57%)
Self or ambulance			
Non-opioid analgesics	673 (44%)	N/A	N/A
Paracetamol	581 (38%)	N/A	N/A
NSAIDs	295 (19%)	N/A	N/A
Opioid analgesics	264 (17%)	N/A	N/A
Anti-emetics	197 (13%)	N/A	N/A
Triptans	87 (6%)	N/A	N/A
Any of the above self- or ambulance drugs	845 (56%)	N/A	N/A

NSAID = non-steroidal anti-inflammatory drug.

N/A = not applicable.

Table 3. Emergency department drug treatment of primary headache

ED drug treatment	Initial and secondary drugs	Initial drugs	Secondary drugs*
Patients	1521	1139	382
None	264 (17%)	N/A	N/A
Non-opioid analgesic	1036 (68%)	853 (56%)	346 (23%)
Paracetamol	876 (58%)	698 (46%)	243 (16%)
NSAIDs	673 (44%)	499 (33%)	223 (15%)
Oral	633 (42%)	476 (31%)	206 (14%)
Parenteral	40 (2.6%)	23 (2%)	17 (1%)
Opioid analgesics, any	495 (33%)	353 (23%)	213 (14%)
Oxycodone	224 (15%)	136 (9%)	111 (7%)
Codeine	214 (14%)	160 (11%)	67 (4%)
Another oral opioid	107 (7%)	68 (4%)	56 (4%)
Any parenteral opioid	58 (4%)	37 (2%)	32 (2%)
Anti-dopaminergic	478 (31%)	307 (20%)	190 (12%)
Prochlorperazine	251 (17%)	150 (10%)	107 (7%)
Metoclopramide	218 (14%)	149 (10%)	74 (5%)
Droperidol	34 (2%)	20 (1%)	14 (0.9%)
Ondansetron	321 (21%)	242 (16%)	97 (6%)
Chlorpromazine infusion	281 (18%)	130 (9%)	160 (11%)
Triptans	46 (3%)	21 (1%)	25 (2%)
Corticosteroids	23 (2%)	9 (0.6%)	14 (0.9%)

NSAID = non-steroidal anti-inflammatory drug.

N/A = not applicable.

* Secondary drugs were given > 30 minutes after the initial drug was administered. It is unknown whether they were used as rescue medication.
